# Rapid behavioral assay using handling test provides breed and sex differences in tameness of chickens

**DOI:** 10.1002/brb3.1394

**Published:** 2019-08-28

**Authors:** Saki Moroi, Kenji Nishimura, Nana Imai, Kyoko Kunishige, Shun Sato, Tatsuhiko Goto

**Affiliations:** ^1^ Department of Life and Food Sciences Obihiro University of Agriculture and Veterinary Medicine Obihiro Japan; ^2^ Agricultural Research Department Animal Research Center Hokkaido Research Organization Sapporo Japan; ^3^ Research Center for Global Agromedicine Obihiro University of Agriculture and Veterinary Medicine Obihiro Japan; ^4^ Present address: Graduate School of Biosphere Science Hiroshima University Higashi‐Hiroshima Japan

**Keywords:** behavior, breed, chickens, heterosis, sex, tameness

## Abstract

**Background:**

Japanese indigenous chicken breeds are often used to improve meat quality rather than broilers in the Jidori industry. There are sometimes severe crowding accidents caused by many birds frightened by environmental stimuli. To prevent the economic loss, the chickens need to be more gentle, tame, and imperturbable.

**Methods:**

In this study, a new handling test for tameness in adult chickens in individual cages was performed with 100 birds from each sex of Shamo, Rhode Island Red, Nagoya, Australorp, and Ukokkei, as well as 10 hens of F_1_ hybrid between Shamo and Rhode Island Red, to measure both active and passive tameness. We counted the number heading toward human hands (heading) and retreating in other directions (avoiding) in both active and passive tameness phases, as well as the number of steps taken (step) during the handling test.

**Results:**

Male chickens exhibited higher avoidance behavior than females. Nagoya females displayed the lowest level of avoidance behavior, which implies passive tameness. In terms of active tameness, a variety of phenotypes can be obtained in different combinations of breed and sex. These results suggested the handling test will be good method for rapid screening of individual differences in tameness. In addition, there were heterosis effects on avoidance and locomotive behaviors. Since F_1_ is often used in the Jidori industry, the breeders should be tested not only for meat production but also for tameness.

**Conclusions:**

In the future, combining both the behavioral screening and the population genomics will establish typical evidence about mechanisms of tameness and domestication in animals.

## Graphical abstract

In this study, a new handling test for tameness in adult chickens in individual cages was performed with 100 birds from each sex of Shamo, Rhode Island Red, Nagoya, Australorp, and Ukokkei, as well as 10 hens of F1 hybrid between Shamo and Rhode Island Red, to measure both active and passive tameness. Male chickens exhibited higher avoidance behavior than females, whereas Nagoya females displayed the lowest level of avoidance behavior, which implies passive tameness. These results suggested the handling test will be good method for rapid screening of individual differences in tameness.

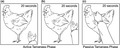

## 1 INTRODUCTION

Chickens were domesticated from jungle fowls (mainly red junglefowl) around areas of South and Southeast Asia (Liu et al., 2006; Peters, Lebrasseur, Deng, & Larson, 2016). After domestication, chickens have been bred worldwide and diversified greatly. There are several hundred chicken breeds in the world, and approximately 50 breeds of chickens in Japan (Tsudzuki, 2003). Chickens provide us food resources such as meat and eggs as well as opportunities for enjoyment of ornamental and behavioral characteristics, which include: long tail feathers, crowing, and aggressive and tameness behaviors (Ekarius, 2007). Productivity of egg and meat has improved markedly in layer and broiler industries, respectively. Of layers, the number of eggs per hen per year improved from 170 eggs in 1925 to as many as 325 eggs in 2006 (Besbes, Tixier‐Boichard, Hoffmann, & Jain, 2007). Havenstein, Ferket, and Qureshi (2003) reported that genetics, nutrition, and other management changes over the last 44 years (from 1957 to 2001) have resulted in broilers that required approximately one‐third time (32 vs. 101 days) and over a threefold decrease in the amount of feed consumed (feed conversion of 1.47 vs. 4.42) to produce an 1,815 g broiler, which means 85%–90% of the increase in broiler growth is contributed by genetic selection. Because the diverse indigenous breeds, layer, and broiler show a wide variety of phenotypes, the genetic basis of preferred traits should be investigated (Goto & Tsudzuki, 2017).

Broilers make up the largest percentage of meat birds produced by the Japanese chicken industry. The broiler has been selected intensely for growth, livability, feed conversion, carcass characteristics, and immune function in the major international breeder companies (Havenstein, Ferket, & Qureshi, 2003). Jidori brand, derived from Japanese indigenous chickens (Tsudzuki, 2003) accounts for approximately 1.5% of the chicken meat market in Japan (Ministry of Agriculture, Forestry and Fisheries of Japan, 2013). The Jidori chickens are mostly hybrids created by crosses between Japanese indigenous breeds (e.g., Shamo and Hinaidori) and American breeds (e.g., White Plymouth Rock and Rhode Island Red) to produce more delicious meat than commercial broilers (F_1_ hybrid between White Cornish and White Plymouth Rock) meat (Tsudzuki, 2003). The aim of the Jidori industry is to provide added value in meat‐producing birds rather than in the development of broilers. Actually, some consumers expect that the Jidori meat is different from broiler meat based on its taste, odor, and texture (Sasaki et al., 2017). In order to achieve making better quality of meat, the Jidori industry makes the best use of genetic and environmental factors, which are as follows: genetically different Japanese indigenous breeds, some nutrient‐added feeds, and opened noncage floor rearing systems. Although each Prefectural Livestock Research Institute tries to improve growth, carcass, and immune traits in Jidori as well as broiler, the Jidori chickens generally grow slower than broiler. However, some benefits found in Jidori meat, for example, Hinai‐jidori chickens showed significantly higher content of arachidonic acid in the thigh meat than broiler (Rikimaru & Takahashi, 2010). In addition, behavioral characteristics are important to improve the Jidori industry. Since the Jidori brand is supported by opened free‐range floor rearing systems mainly, there are sometimes severe crowding accidents where hundreds of birds in the flock die due to traumatic asphyxia caused by crushing from many birds frightened by environmental stimuli, such as noise pollution and thunder. The Nagoya breed, which is often used in the Jidori brand, has been reported as a cowardly bird (Hong, Inoue‐Murayama, Nakamura, Nagao, & Ito, 2008). In order to prevent economic loss by the accidents, the chickens are required to be more gentle, tame, and imperturbable toward humans' handling and environmental stimuli in behavioral traits.

Animal tameness has been investigated using experimentally domesticated animal resources that are foxes, rats, mice, and red junglefowls (Albert et al., 2011; Katajamaa, Larsson, Lundberg, Sorensen, & Jensen, 2018; Matsumoto et al., 2017; Wang et al., 2018). Behavioral tests have been established for measuring fox response to a human observer using a standard four‐step test in their home cages (Nelson et al., 2017), rat response of tameness/aggressiveness when confronted with a gloved human hand in a test cage (Albert et al., 2008), and bird fear response toward humans in an arena measuring 100 × 300 × 210 cm (Agnvall, Jongren, Strandberg, & Jensen, 2012). Because tameness is defined as increased interaction of animals with humans and Price (2002) stated that tameness has two behavioral components which are “a measure of the extent to which an individual is reluctant to avoid or motivated to approach humans,” we established tameness tests for mice to measure active approaches to humans (active tameness) and a reduction in the avoidance of humans (passive tameness) separately (Goto, Tanave, Moriwaki, Shiroishi, & Koide, 2013; Nagayama et al., 2018). Since there is no example of a behavioral test to measure these kinds of tame behaviors using adult chickens in individual cages, we will introduce a handling test for tameness of adult chickens in this study. Breeding of Jidori often utilizes grand‐parental and parental stocks in individual cages in the National Livestock Center and Prefectural Livestock Farms in Japan. If a suitable method to test tame behaviors of adult chickens is developed, breeding toward tameness of Jidori can be applied in the future. Since there is still no clear correlation between reactions to humans (tameness) and to environmental stimuli, the birds selected for tameness may show not only reduction in anxiety to humans but also imperturbable toward environmental stimuli in opened free‐range floor rearing systems. Moreover, revealing the genetic basis of tame behavior is crucial to understanding future application to the livestock industry and the history of animal domestication (Goto, Matsumoto, Tanave, & Koide, 2015).

In this study, we try to establish a handling test for tame behavior of adult chickens in their home‐cage. The objective of this study is to investigate breed and sex differences in tame behaviors by the handling test. This will be a first step in evaluating and understanding tameness in adult chickens.

## 2 MATERIALS AND METHODS

### 2.1 Animals

Five breeds and one F_1_ hybrid of chickens were used in the Animal Research Center, Agricultural Research Department, Hokkaido Research Organization, Japan. A total of 100 birds from each sex of Shamo (SHA; *n* = 10), Rhode Island Red (RIR; *n* = 10), Nagoya (NGY; *n* = 10), Australorp (AUS; *n* = 10), and Ukokkei (UKO; *n* = 10) were investigated. In addition, we analyzed only hens of F_1_ hybrid (F_1_; *n* = 10) based on a cross between SHA males and RIR females. The chickens were reared under the photoperiod cycle of 16‐hr light and 8‐hr dark with free access to diets and water in the individual cages. Management was performed on according to the rules of Standards Related to the Care and Management of Experimental Animals (Prime Ministers' Office, Japan, 1980) and the Guide for the Use of Experimental Animals in Universities (The Ministry of Education, Science, Sports, and Culture, Japan, 1987).

### 2.2 Tameness test

Based on the behavioral tests for tameness in foxes (Nelson et al., 2017), rats (Albert et al., 2008), and mice (Goto et al., 2013; Nagayama et al., 2018), we designed a handling test that measured active approaches to humans (active tameness) and a reduction in the avoidance of humans (passive tameness) in adult chickens. The handling test was performed using adult chickens (>23 weeks of age) in their home cages. The handling test was set for 60 s in total. There were two phases, which are the active tameness phase (first 40 s) and the passive tameness phase (last 20 s).

**Figure 1:**
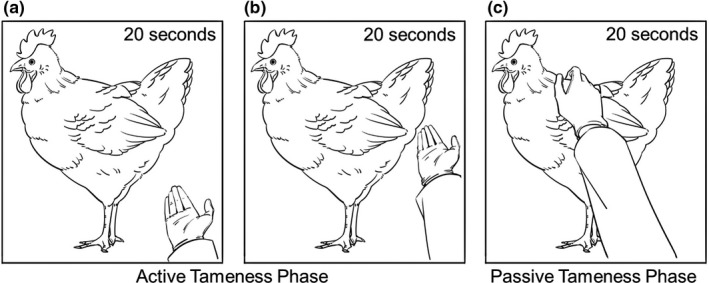
Schematic representations of handling test for adult chickens in cage. Total duration of tameness test is 1 min. (a) In first 20 s of active tameness phase, experimenter's hand stays near the entrance of the cage. (b) In the next 20 s of active tameness phase, experimenter's hand moves to the far end and remains. (c) Just after finishing active tameness phase, passive tameness phase starts. For 20 s, experimenter keeps on stroking the back of chicken in frequency of once per 2 s

At the active tameness phase of the handling test, the experimenter's hand stayed in the cage in order to evaluate active tameness of the chicken. The active tameness phase consisted of two steps. The first step was to keep the experimenter's hand near the entrance of the cage for 20 s (Figure 1a). After that, the experimenter's hand moved to the far end of the cage and stayed for 20 s, as the second step (Figure 1b). Immediately after the active tameness phase, the passive tameness phase was started. At the passive tameness phase of the handling test, the experimenter stroked the backs of the chickens in order to evaluate passive tameness of the chickens (Figure 1c). The frequency of stroking was set at approximately once per 2 s. After 20 s of passive tameness phase, the experimenter removed his hand from the cage.

We focused on direction and frequency of moving away in reactive behaviors during the handling test. If the chicken shows some interest and/or positive reaction toward the experimenter's hand, the number of times heading toward the hand is expected to increase along with being high in explorative behavior. Conversely, if the chicken expresses less interest and/or negative reactions toward the experimenter's hand, the number of times heading toward the hand is expected to decrease along with being high in active avoidance. Therefore, we measured both the number of times a chicken heads toward the experimenter's hand (shown as heading) and the number of times a chicken avoids in other directions (shown as avoiding), as well as number of steps taken (shown as step) for locomotive activity. Behavioral analysis was performed in real time by two observers. An observer counted numbers heading and avoiding in active and passive tameness phases separately. The other observer counted number of steps during the handling test. Behaviors were recorded by a video camera (iVIS HF M41, Canon, Japan) for the purpose of confirmation.

### 2.3 Statistics

In order to test effects according to breed and sex, we used 100 chickens in total in RIR, SHA, NGY, UKO, and AUS (*n* = 10 in each breed and sex). Main effects by breed and sex and their interaction effect were tested by two‐way analysis of variance (ANOVA). If there is significant difference (*p* < .05), a post hoc test was performed by Tukey's HSD test. In addition, in order to test the heterosis effect on behavioral traits, one‐way ANOVA was carried out with the data of F_1_ hybrid hens derived from a cross between SHA and RIR (*n* = 10), and hens of SHA and RIR (*n* = 10 in each breed). Pearson's correlations among behavioral traits were calculated. Data were shown in mean ± standard deviation. Statistical analyses were conducted by R software (R Core Team, 2018).

## 3 RESULTS

### 3.1 Active tameness phase of the handling test

**Figure 2:**
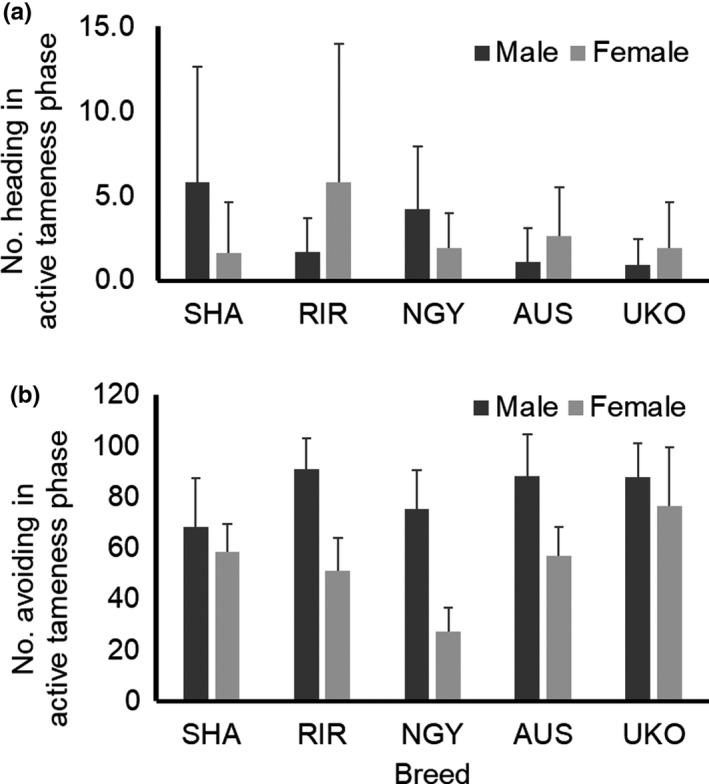
Behavioral traits in active tameness phase of handling test. Bar indicates standard deviation. Shamo (SHA), Rhode Island Red (RIR), Nagoya (NGY), Australorp (AUS), and Ukokkei (UKO) were used. (a) Means of number heading in active tameness phase are shown in each breed and sex. Two‐way ANOVA reveals significant breed × sex interaction effect (*p* < .05), but not main effects by breed and sex. (b) Means of number of avoiding in active tameness phase are shown in each breed and sex. Significant main effects and interaction effect by breed and sex (*p* < .05) are found by two‐way ANOVA

In the number heading at the active tameness phase (Figure 2a), two‐way ANOVA revealed no main effects by breed (*F*_4,90_ = 1.239, *p* = .300) or sex (*F*_1,90_ = 0.001, *p* = .982), but a significant showing of interaction effects by breed × sex (*F*_4,90_ = 2.880, *p* = .027). Although SHA and NGY showed that females were lower than males, the opposite relationship was seen in RIR, AUS, and UKO. This meant the combination of breed and sex influenced phenotypic variations in the heading trait at the active tameness phase. Mean values in all groups were low (from 0.9 to 5.8), which implying that the chickens showed low levels in active approaches to humans (active tameness).

In terms of avoiding at the active tameness phase (Figure 2b), there were significant main effects by breed (*F*_4,90_ = 10.563, *p* = 4.62E−07) and sex (*F*_1,90_ = 78.795, *p* = 6.22E−14) and their interaction effect by breed × sex (*F*_4,90_ = 5.907, *p* = .0003). This avoiding trait was thought to indicate a chicken's tendency to avoid the mild stimulus, the presence of a human hand. Males exhibited a higher number avoiding in other directions from a human hand than the females. RIR and SHA were the highest and lowest mean values in males, respectively, whereas UKO and NGY were the highest and lowest of those in female. Since NGY females were especially lower than the others, NGY females showed the lowest level of avoidance of humans (passive tameness) from mild stimuli by human hands.

### 3.2 Passive tameness phase of the handling test

**Figure 3:**
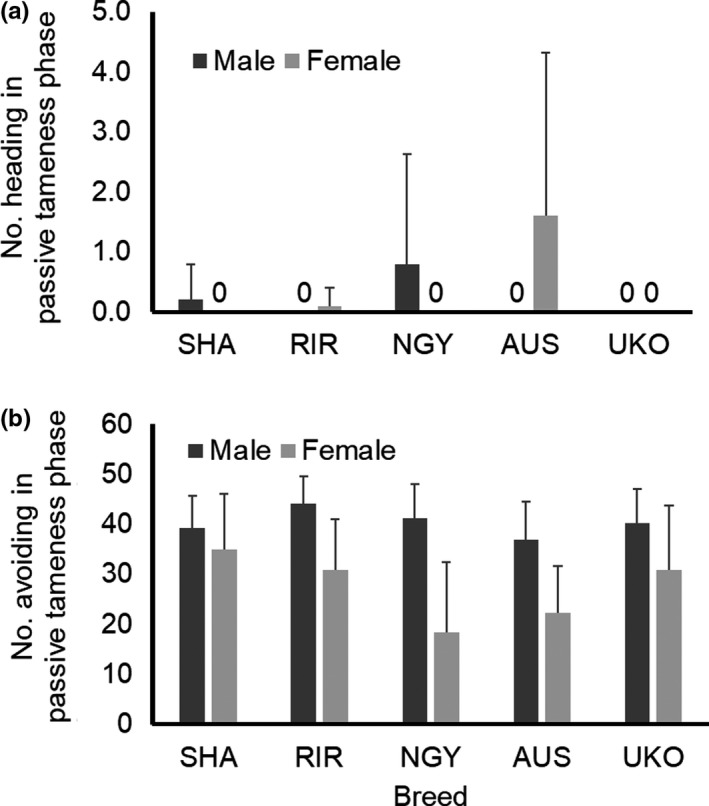
Behavioral traits in passive tameness phase of handling test. Bar indicates standard deviation. Shamo (SHA), Rhode Island Red (RIR), Nagoya (NGY), Australorp (AUS), and Ukokkei (UKO) were used. “0” means all individuals represent zero value. (a) Means of number of heading in passive tameness phase are shown in each breed and sex. Two‐way ANOVA reveals significant breed × sex interaction effect (*p* < .05), but not main effects. (b) Means of number avoiding in passive tameness phase are shown in each breed and sex. Two‐way ANOVA reveals significant main effects by breed and sex (*p* < .05), whereas no interaction effect is found

Two‐way ANOVA indicated no significant main effects by breed (*F*_4,90_ = 1.792, *p* = .1373) or sex (*F*_1,90_ = 0.392, *p* = .5328), but a significant interaction effect by breed × sex (*F*_4,90_ = 3.152, *p* = .0179) in the number heading at the passive tameness phase (Figure 3a). The heading trait was thought to be active tameness under the severer stimulus, forced handling by a human. Mean values ranged from 0 to 1.6, and all chickens in the six groups indicated zero value. This implied that the chickens do not actively approach a human hand under the condition of forced handling by a human.

In terms of avoiding at the passive tameness phase (Figure 3b), two‐way ANOVA revealed significant main effects by breed (*F*_4,90_ = 3.097, *p* = .0194) and sex (*F*_1,90_ = 41.478, *p* = 5.71E−09), but not by their interaction effect (*F*_4,90_ = 2.343, *p* = .0608). The avoiding trait was thought to show a chicken's tendency to avoid forced handling by a human. Males showed higher number avoiding than females. NGY and AUS indicated lower mean values than the others, especially in females, which implying these birds were thought to have a high level of passive tameness.

### 3.3 Locomotive activity in handling test

**Figure 4:**
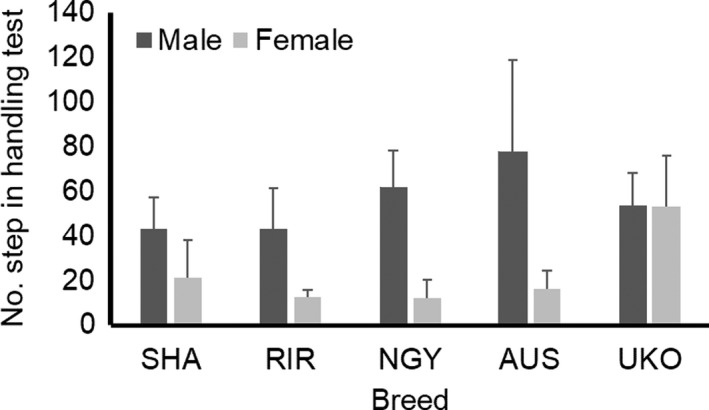
Locomotive activity (step) in handling test. Bar indicates standard deviation. Shamo (SHA), Rhode Island Red (RIR), Nagoya (NGY), Australorp (AUS), and Ukokkei (UKO) were used. Means of number of steps during handling test are shown in each breed and sex. Two‐way ANOVA reveals significant main effects and interaction effect by breed and sex (*p* < .05)

In number of steps taken during the handling test (Figure 4), there were significant main effects by breed (*F*_4,90_ = 5.530, *p* = .0005) and sex (*F*_1,90_ = 67.106, *p* = 1.64E−12) and their interaction effect (*F*_4,90_ = 7.142, *p* = 4.85E−05). The step trait indicated total amount of locomotive activity. Males basically showed higher activity than females. Females in RIR, NGY, and AUS indicated lower activity, whereas males in NGY and AUS showed higher activity. On the other hand, UKO indicated comparable levels of activity between males and females.

### 3.4 Phenotypic correlations among behavioral traits

**Figure 5:**
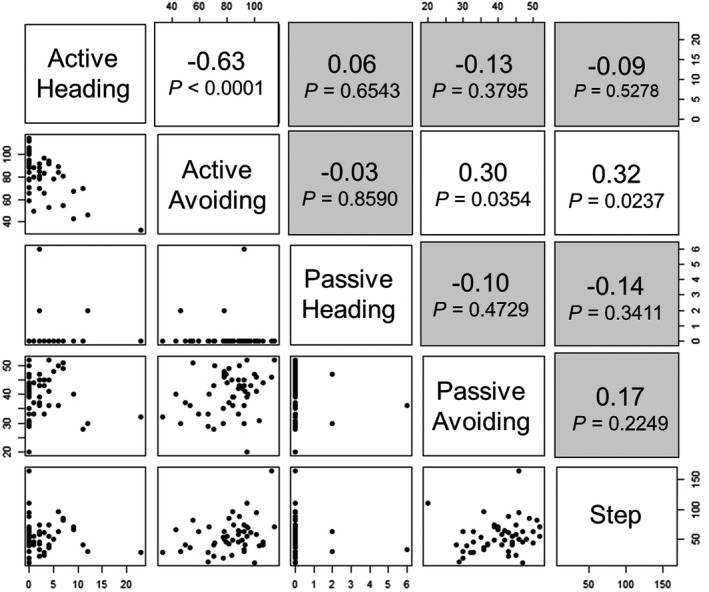
Scatter diagrams and phenotypic correlations between behavioral traits in male. Data from five breeds (*n* = 50) are plotted on each panel on the lower left side. Pearson's correlation coefficients between two different traits are shown on the upper right side. The cells showing no significance are expressed in gray background (*p* > .05)

**Figure 6:**
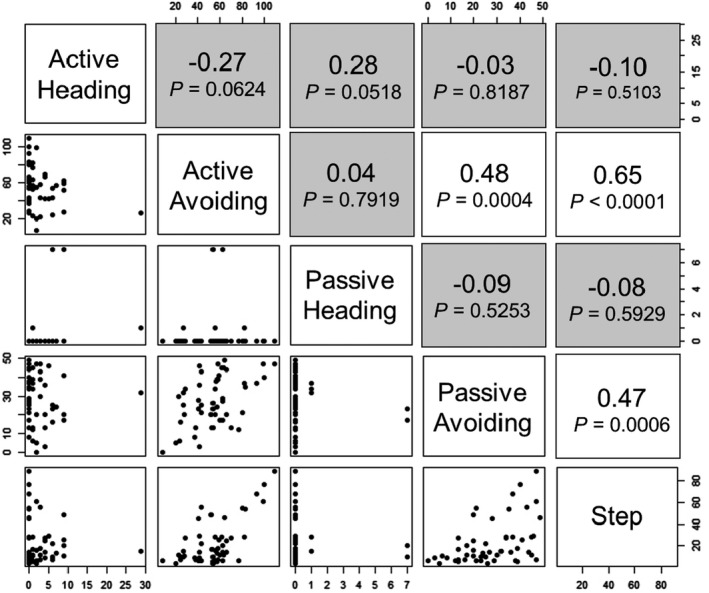
Scatter diagrams and phenotypic correlations between behavioral traits in female. Data from five breeds (*n* = 50) are plotted on each panel on the lower left side. Pearson's correlation coefficients between two different traits are shown on the upper right side. Gray colored cells indicate no significance (*p* > .05)

In this study, five behavioral traits were evaluated with males and females from five breeds (*n* = 100). In three traits, significant sex effects were detected. Therefore, we calculated phenotypic correlations in each sex in order to compare the relationships between the different behavioral traits. Pearson's correlation coefficients among the five traits in males and females were shown in Figures 5 and 6. There were positive phenotypic correlations between avoiding at active tameness phase and avoiding at passive tameness phase (*r* = .30 and .48 in male and female, respectively) and between avoiding at active tameness phase and number of steps during handling test (*r* = .32 and.65 in male and female, respectively). In males, there was a negative correlation (*r* = −.63) between heading and avoiding at the active tameness phase. On the other hand, in females, a positive correlation (*r* = .47) was seen between avoiding at the passive tameness phase and number of steps counted during the handling test. In the remaining trait combinations, correlation coefficients were statistically comparable to zero (*p* > .05). These indicated there is a similar tendency of phenotypic correlations in males and females. In the case of high value in avoiding, locomotive activity and active approach behavior will show increases and decreases, respectively.

### 3.5 Heterosis effect

**Figure 7:**
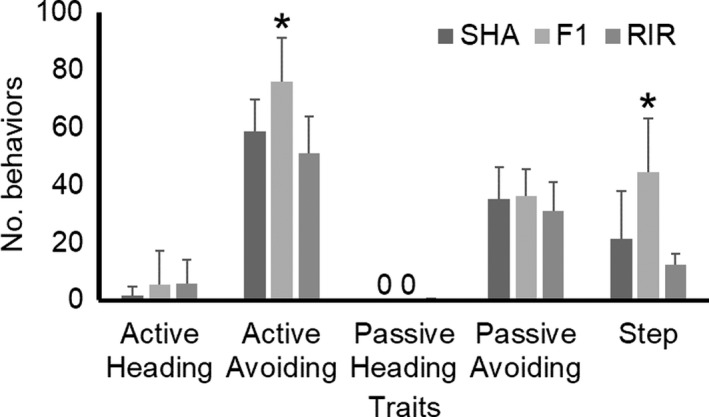
Heterosis effect on behavioral traits in handling test. Bar indicates standard deviation. Females in Shamo (SHA), Rhode Island Red (RIR), and F_1_ hybrid (F_1_) based on a cross between SHA males and RIR females were used. “0” means all individuals represent zero value. One‐way ANOVA reveals significant effects on heterosis in avoiding at active tameness phase and locomotive activity during handling test (*p* < .05) as indicated by an asterisk above F_1_ hybrid value. There is no heterosis effect in the other traits

In order to evaluate the heterosis effects on behavioral traits, we analyzed female data from SHA, RIR, and their F_1_ hybrid. In Figure 7, one‐way ANOVA revealed significant effects by breed in avoiding at active tameness phase (*F*_2,27_ = 8.624, *p* = .0013) and step (*F*_2,27_ = 11.290, *p* = .0003). Tukey's HSD tests indicated there were significant differences between F_1_ hybrid and their parental breeds (*p* < .05), but no difference among parental breeds (*p* > .05). The F_1_ hybrid had higher avoidance and locomotive behaviors than those of the parental breeds.

## 4 DISCUSSION

The present study aimed to make a new handling test to quantify animal tameness in adult chickens reared in individual cages. Male chickens indicated higher avoidance behavior from a human hand than females. NGY female had the lowest level of avoidance behavior, which implies passive tameness. In terms of active tameness, a breed × sex interaction effect was observed, which indicated a variety of phenotypes can be obtained in different combinations of breed and sex. There was a type of individual that showed active tame behavior toward humans, whereas no individual expressed aggressive (nontame) behavior toward humans in this study. These results suggested that the handling test has a potential to reveal characteristics of animal tameness, such as active tameness (“motivation to approach humans”) and passive tameness (“reluctant to avoid humans”) behaviors, even though the duration of the handling test is only one minute per individual. Thus, the handling test will be one of the optimal methods for rapid screening of individual differences in chickens' tame behavior toward humans.

Regarding tameness, our previous genetic correlation analyses with 17 wild‐derived and laboratory strains of mice revealed evidence that when a higher level of passive tame behavior is observed, active tame behavior tends to be high (Goto et al., 2013). Furthermore, comparative analyses between wild and domestic mice revealed that the heritable behavioral characteristics, reluctance to avoid humans but not motivation to approach humans, will be the key for domestication of mice (Goto et al., 2013). There is massive evidence that long‐term selection of tameness significantly increased the tame behavior in foxes, rats, and mice (Matsumoto et al., 2017; Trut, 1999). Agnvall et al. (2012) have estimated 17% of heritability for fear‐related behavior in the fear of humans test with chickens' wild ancestor, red junglefowl (RJF). Given that animal tameness is defined some extent to increase interaction of animals with humans, tameness is involved with many behavioral characters including anxiety, fear, and novelty seeking. There are some hypotheses that (a) animals with higher tameness show low levels of anxiety and fearfulness toward not only humans but also other external stimuli and (b) high‐tameness animals show a high level of novelty seeking toward humans and the other stimuli. In the Jidori brands, there are sometimes severe crowding accidents where hundreds of birds in the flock die due to environmental stimuli, such as thunder. There are proactive and reactive coping styles in animals. Proactive animals show a more sympathetic stress activation (flight/fight), although reactive animals often respond to a stressful situation by a higher parasympathetic stress activation (withdrawal reaction; Favati, Leimar, & Løvlie, 2014). Proactive/reactive animals tend to show higher/lower in aggressive and active avoidance, and lower/higher in HPA axis reactivity, respectively. Moreover, reactive animals are more flexible and react to environmental stimuli (Koolhaas et al., 1999). Further studies are needed to understand the relationship between reactions to humans (tameness) and the other behavioral and physiological traits, including reactivity to environmental stimuli, as well as for productivity of eggs and meat.

Natt, Agnvall, and Jensen (2014) have reported substantial sex differences in chicken behavior with many RJF, using a battery of behavioral tests for phenotyping different aspects of fear, exploration, and sociability. In open field tests and foraging and exploration tests, males displayed less fear and less exploration than females. On the other hand, in predator tests and tonic immobility tests, females were more fearful than males. They concluded that females were more explorative and prone to forage, but more fearful in their reactions to various stressful stimuli in RJF (Natt et al., 2014). Nakasai et al. (2013) also indicated sexual differences in fear response via tonic immobility in Tosa‐Jidori chicks, which indicated females were more fearful than males. Conversely, our handling test revealed that females exhibited more passive tameness than males. This evidence may imply that female chickens in this study accepted handling (passive tameness) with some levels of fear‐related behavior, because life‐threatening events tend to induce the animals to both fight or flight and freezing (Peres, Goncalves, & Peres, 2009). Regarding this, male chickens in this study showed higher levels of flight response from handling than females. Although it is difficult to discriminate clearly between low levels of reaction to handling (passive tameness) or freezing in response to handling (nontameness), our tameness test can evaluate to some extent avoidance (flight) of human handling. Given that the crowding accidents are caused by crushing birds frightened by environmental stimuli, low levels of reaction to the stimuli may be useful behavioral characteristics for preventing the accidents and reducing economic losses in the Jidori industry.

In domestic chickens, there was an effect on fear response by breed using tonic immobility test for chicks of NGY and White Leghorn (WL), which indicated WL showed relatively lower fear levels than NGY (Abe, Nagao, Nakamura, & Inoue‐Murayama, 2013). Tomonaga et al. (2007) investigated stress responses in chicks using both meat‐type and layer‐type NGY, which are selected according to two directions over a few decades and found that meat‐type chicks indicated bolder behavior under isolation‐induced stress than the layer‐type of NGY. In this study, we analyzed meat‐type NGY, which is a different strain from the previous ones (Tomonaga et al., 2007), and found that NGY females are less likely to avoid handling. These results imply that behaviors in chicks will be a good indicator to estimate those behaviors in the adult stage. Favati, Zidar, Thorpe, Jensen, and Lovlie (2016) have investigated ontogeny of personality traits in RJF from chick (4 weeks of age) to adult (40 weeks of age). They performed three different personality tests, which are as follows: the novel arena test, the tonic immobility test, and the predator test; and found that the adult personality can only to some extent be predicted early in life (Favati et al., 2016). Although there are some studies using chicks in Japanese breeds, there may be low correlations among them. In the future, behavioral relationships between chicks and adult chickens should be analyzed more using many Japanese breeds of chickens. For breeding tameness, it may be effective to use behavioral phenotypes at several stages.

This study revealed heterosis effects on avoidance and locomotive behaviors, indicating that F_1_ hybrid was significantly higher than those of parental breeds. In the Jidori industry of Japan, F_1_ hybrids are often used to enhance performance in growth, meat production, and disease resistance using the benefits of a heterosis effect. It is generally considered that hybrids combine the best traits of both parental breeds and thus exhibit better performance in several organisms (Comings & MacMurray, 2000). Heterosis for fear of humans has been reported using Mule duck from a cross between a Muscovy duck and a Pekin duck (Faure, Val‐Laillet, Guy, Bernadet, & Guemene, 2003). Arnaud et al. (2008) have investigated more detailed data using those ducks and found significant heterosis effects on behavioral responses and basal adrenal activity that is hybrids showed higher fear responses to human and higher basal level of corticosterone compared to the parent genotypes. This evidence indicated that there will be a possible common mechanism underlying heterosis of response to humans in not only avian but also other livestock animals. Since it is very likely that the presence or the absence of molecular heterosis can also depend on the genetic background (Comings & MacMurray, 2000), the Jidori brand should be tested not only for growth, meat production, and disease‐related performance but also for behavior toward humans (tameness) and fear response from environmental stimuli with F_1_ hybrids from several kinds of crosses, as well as the parental breeds themselves.

As Price (2002) indicated that tameness is defined “reluctant to avoid or motivated to approach humans,” a low level of fearfulness will be common behavioral characteristics in domestic animals. Actually, Campler, Jongren, and Jensen (2009) have reported that the fear response of WL is less than chicken's wild ancestor, RJF in four different behavioral tests including fear of humans test, which suggested selection for low fearfulness has been an important element of domestication. Agnvall, Katajamaa, Altimiras, and Jensen (2015) have selected RJF for high or low fear of humans during six generations and revealed that low‐fear birds showed higher basal metabolic rate, feed efficiency, and boldness in novel object test. Selection of tameness brought many of phenotypic changes in tissue size (adrenal gland and spleen), serum and brain biochemical components (corticosterone, glucose, serotonin, and taurine) in rats, and physical changes (coat color, floppy ears, and rolled tails) in foxes, along with the Belyaev's hypothesis (Albert et al., 2008; Trut, 1999). There are massive efforts to understand the genetic basis of animal domestication using comparative population genomics with chickens, dogs, rabbits, cats, and their wild ancestors (Axelsson et al., 2013; Carneiro et al., 2014; Montague et al., 2014; Rubin et al., 2010). Since animal tameness is a key feature for domestication, further researches are required to reveal the genetic basis regulating the phenotypic variation in tameness. In this study, our efficient handling test can reveal phenotypic differences by breed and sex in tameness of adult chickens. Osman et al. (2006) revealed a wide variety of genetic backgrounds among SHA, NGY, UKO, and RIR. In the future, we would like to analyze the tameness with cross experiments to reveal whether behavioral changes among breeds are due to overall genomic differences or some specific genetic differences related to tameness. Combining both the behavioral screening and the population genomics will find types of evidence about the mechanism of tameness and domestication in animals.

In conclusion, we established a new handling test to evaluate tame behavior of adult chickens and revealed breed and sex differences and heterosis effects. The Japanese Jidori industry, which aimed to increase value‐added meat production should pay attention to behavioral responses toward humans in the future. This study is a first step to conduct genetic study of tame behavior in chickens.

## CONFLICT OF INTEREST

The authors declare no conflict of interest.

## Data Availability

The data that support the findings of this study are available from the corresponding author upon reasonable request.

## References

[brb31394-bib-0001] Abe, H. , Nagao, K. , Nakamura, A. , & Inoue‐Murayama, M. (2013). Differences in responses to repeated fear‐relevant stimuli between Nagoya and White Leghorn chicks. Behavioural Processes, 99, 95–99. 10.1016/j.beproc.2013.07.004 23860281

[brb31394-bib-0002] Agnvall, B. , Jongren, M. , Strandberg, E. , & Jensen, P. (2012). Heritability and genetic correlations of fear‐related behavior in Red Junglefowl‐possible implications for early domestication. PLoS ONE, 7, e35162. 22536354 10.1371/journal.pone.0035162 PMC3334967

[brb31394-bib-0003] Agnvall, B. , Katajamaa, R. , Altimiras, J. , & Jensen, P. (2015). Is domestication driven by reduced fear of humans? Boldness, metabolism and serotonin levels in divergently selected red junglefowl (*Gallus gallus*). Biology Letters, 11, 20150509. 26382075 10.1098/rsbl.2015.0509 PMC4614427

[brb31394-bib-0004] Albert, F. W. , Hodges, E. , Jensen, J. D. , Besnier, F. , Xuan, Z. , Rooks, M. , … Pääbo, S. (2011). Targeted resequencing of a genomic region influencing tameness and aggression reveals multiple signals of positive selection. Heredity, 107, 205–214. 10.1038/hdy.2011.4 21304545 PMC3183948

[brb31394-bib-0005] Albert, F. W. , Shchepina, O. , Winter, C. , Römpler, H. , Teupser, D. , Palme, R. , … Pääbo, S. (2008). Phenotypic differences in behavior, physiology and neurochemistry between rats selected for tameness and for defensive aggression towards humans. Hormones and Behavior, 53, 413–421. 10.1016/j.yhbeh.2007.11.010 18177873

[brb31394-bib-0006] Arnaud, I. , Mignon‐Grasteau, S. , Larzul, C. , Guy, G. , Faure, J.‐M. , & Guémené, D. (2008). Behavioural and physiological fear responses in ducks: Genetic cross effects. Animal, 2, 1518–1525. 10.1017/S1751731108002784 22443910

[brb31394-bib-0007] Axelsson, E. , Ratnakumar, A. , Arendt, M.‐L. , Maqbool, K. , Webster, M. T. , Perloski, M. , … Lindblad‐Toh, K. (2013). The genomic signature of dog domestication reveals adaptation to a starch‐rich diet. Nature, 495, 360–364. 10.1038/nature11837 23354050

[brb31394-bib-0008] Besbes, B. , Tixier‐Boichard, M. , Hoffmann, I. , & Jain, G. L. (2007). Future trends for poultry genetic resources In Proceedings of the International Conference of Poultry in the 21st Century: Avian Influenza and Beyond. Bankok, 5–7 November 2007. Retrieved from http://www.fao.org/waicent/FAOINFO/Agricult/againfo/home/events/bangkok2007/docs/part1/1_8.pdf

[brb31394-bib-0009] Campler, M. , Jongren, M. , & Jensen, P. (2009). Fearfulness in red junglefowl and domesticated White Leghorn chickens. Behavioural Processes, 81, 39–43. 10.1016/j.beproc.2008.12.018 19154782

[brb31394-bib-0010] Carneiro, M. , Rubin, C.‐J. , Di Palma, F. , Albert, F. W. , Alfoldi, J. , Barrio, A. M. , … Andersson, L. (2014). Rabbit genome analysis reveals a polygenic basis for phenotypic change during domestication. Science, 345, 1074–1079. 10.1126/science.1253714 25170157 PMC5421586

[brb31394-bib-0011] Comings, D. E. , & MacMurray, J. P. (2000). Molecular heterosis: A review. Molecular Genetics and Metabolism, 71, 19–31. 10.1006/mgme.2000.3015 11001792

[brb31394-bib-0012] Ekarius, C. (2007). Storey's illustrated guide to poultry breeds. North Adams, MA: Storey Publishing.

[brb31394-bib-0013] Faure, J. M. , Val‐Laillet, D. , Guy, G. , Bernadet, M. D. , & Guemene, D. (2003). Fear and stress reactions in two species of duck and their hybrid. Hormones and Behavior, 43, 568–572. 10.1016/S0018-506X(03)00062-X 12799174

[brb31394-bib-0014] Favati, A. , Leimar, O. , & Løvlie, H. (2014). Personality predicts social dominance in male domestic fowl. PLoS ONE, 9, e103535 10.1371/journal.pone.0103535 25072296 PMC4114777

[brb31394-bib-0015] Favati, A. , Zidar, J. , Thorpe, H. , Jensen, P. , & Lovlie, H. (2016). The ontogeny of personality traits in the red junglefowl, *Gallus gallus* . Behavioral Ecology, 27, 484–493.

[brb31394-bib-0016] Goto, T. , Matsumoto, Y. , Tanave, A. , & Koide, T. (2015). Genetic analyses for tame behavior in animals: Exploration of genetic loci affecting animal domestication. Journal of Animal Genetics, 43, 3–11 (In Japanese). 10.5924/abgri.43.3

[brb31394-bib-0017] Goto, T. , Tanave, A. , Moriwaki, K. , Shiroishi, T. , & Koide, T. (2013). Selection for reluctance to avoid humans during the domestication of mice. Genes, Brain and Behavior, 12, 760–770. 10.1111/gbb.12088 PMC4282115 24034605

[brb31394-bib-0018] Goto, T. , & Tsudzuki, M. (2017). Genetic mapping of quantitative trait loci for egg production and egg quality traits in chickens: A review. Journal of Poultry Science, 54, 1–12. 10.2141/jpsa.0160121 PMC7477176 32908402

[brb31394-bib-0019] Havenstein, G. B. , Ferket, P. R. , & Qureshi, M. A. (2003). Growth, livability, and feed conversion of 1957 versus 2001 broilers when fed representative 1957 and 2001 broiler diets. Poultry Science, 82, 1500–1508. 10.1093/ps/82.10.1500 14601725

[brb31394-bib-0020] Havenstein, G. B. , Ferket, P. R. , & Qureshi, M. A. (2003). Carcass composition and yield of 1957 versus 2001 broilers when fed representative 1957 and 2001 broiler diets. Poultry Science, 82, 1509–1518. 10.1093/ps/82.10.1509 14601726

[brb31394-bib-0021] Hong, K. W. , Inoue‐Murayama, M. , Nakamura, A. , Nagao, K. , & Ito, S. (2008). Characterization of two microsatellites in chicken monoamine oxidase A. Animal Science Journal, 79, 641–643. 10.1111/j.1740-0929.2008.00575.x

[brb31394-bib-0022] Katajamaa, R. , Larsson, L. H. , Lundberg, P. , Sorensen, I. , & Jensen, P. (2018). Activity, social and sexual behaviour in Red Junglefowl selected for divergent levels of fear of humans. PLoS ONE, 13, e0204303 10.1371/journal.pone.0204303 30256834 PMC6157887

[brb31394-bib-0023] Koolhaas, J. M. , Korte, S. M. , De Boer, S. F. , Van Der Vegt, B. J. , Van Reenen, C. G. , Hopster, H. , … Blokhuis, H. J. (1999). Coping styles in animals: Current status in behavior and stress‐physiology. Neuroscience and Biobehavioral Reviews, 23, 925–935. 10.1016/S0149-7634(99)00026-3 10580307

[brb31394-bib-0024] Liu, Y.‐P. , Wu, G.‐S. , Yao, Y.‐G. , Miao, Y.‐W. , Luikart, G. , Baig, M. , … Zhang, Y.‐P. (2006). Multiple maternal origins of chickens: Out of the Asian jungles. Molecular Phylogenetics and Evolution, 38, 12–19. 10.1016/j.ympev.2005.09.014 16275023

[brb31394-bib-0025] Matsumoto, Y. , Goto, T. , Nishino, J. O. , Nakaoka, H. , Tanave, A. , Takano‐Shimizu, T. , … Koide, T. (2017). Selective breeding and selection mapping using a novel wild‐derived heterogeneous stock of mice revealed two closely‐linked loci for tameness. Scientific Reports, 7, 4607 10.1038/s41598-017-04869-1 28676693 PMC5496859

[brb31394-bib-0026] Montague, M. J. , Li, G. , Gandolfi, B. , Khan, R. , Aken, B. L. , Searle, S. M. J. , … Warren, W. C. (2014). Comparative analysis of the domestic cat genome reveals genetic signatures underlying feline biology and domestication. Proceedings of the National Academy of Sciences of the United States of America, 111(48), 17230–17235. 10.1073/pnas.1410083111 25385592 PMC4260561

[brb31394-bib-0027] Nagayama, H. , Matsumoto, Y. , Tanave, A. , Nihei, M. , Goto, T. , & Koide, T. (2018). Measuring active and passive tameness separately in mice. Journal of Visualied Experiments, 138, e58048 10.3791/58048 PMC6126707 30148490

[brb31394-bib-0028] Nakasai, E. , Tanizawa, H. , Takawaki, M. , Yanagita, K. , Kawakami, S.‐I. , Oka, T. , … Bungo, T. (2013). Age‐dependent change of tonic immobility response in chicks of a native Japanese chicken breed, Tosa‐Jidori. Journal of Poultry Science, 50, 321–325. 10.2141/jpsa.0130018

[brb31394-bib-0029] Natt, D. , Agnvall, B. , & Jensen, P. (2014). Large sex differences in chicken behavior and brain gene expression coincide with few differences in promoter DNA‐methylation. PLoS ONE, 9, e96376 10.1371/journal.pone.0096376 24782041 PMC4004567

[brb31394-bib-0030] Nelson, R. M. , Temnykh, S. V. , Johnson, J. L. , Kharlamova, A. V. , Vladimirova, A. V. , Gulevich, R. G. , … Kukekova, A. V. (2017). Genetics of interactive behavior in silver foxes (*Vulpes vulpes*). Behavior Genetics, 47, 88–101. 10.1007/s10519-016-9815-1 27757730

[brb31394-bib-0031] Osman, S. A. M. , Sekino, M. , Nishihata, A. , Kobayashi, Y. , Takenaka, W. , Kinoshita, K. , … Tsudzuki, M. (2006). The genetic variability and relationships of Japanese and foreign chickens assessed by microsatellite DNA profiling. Asian‐Australasian Journal of Animal Science, 19, 1369–1378. 10.5713/ajas.2006.1369

[brb31394-bib-0032] Peres, J. F. P. , Goncalves, A. L. , & Peres, M. F. P. (2009). Psychological trauma in chronic pain: Implications of PTSD for fibromyalgia and headache disorders. Current Pain and Headache Reports, 13, 350–357. 10.1007/s11916-009-0057-2 19728960

[brb31394-bib-0033] Peters, J. , Lebrasseur, O. , Deng, H. , & Larson, G. (2016). Holocene cultural history of Red jungle fowl (*Gallus gallus*) and its domestic descendant in East Asia. Quaternary Science Reviews, 142, 102–119. 10.1016/j.quascirev.2016.04.004

[brb31394-bib-0034] Price, E. O. (2002). Animal domestication and behavior. New York, NY: CABI Publishing.

[brb31394-bib-0035] R Core Team (2018). R: A language and environment for statistical computing. Vienna, Austria: R Foundation for Statistical Computing Retrieved from https://www.R-project.org/

[brb31394-bib-0036] Rikimaru, K. , & Takahashi, H. (2010). Evaluation of the meat from Hinai‐jidori chickens and broilers: Analysis of general biochemical components, free amino acids, inosine 5′‐monophosphate, and fatty acids. The Journal of Applied Poultry Research, 19, 327–333. 10.3382/japr.2010-00157

[brb31394-bib-0037] Rubin, C.‐J. , Zody, M. C. , Eriksson, J. , Meadows, J. R. S. , Sherwood, E. , Webster, M. T. , … Andersson, L. (2010). Whole‐genome resequencing reveals loci under selection during chicken domestication. Nature, 464, 587–591. 10.1038/nature08832 20220755

[brb31394-bib-0038] Sasaki, K. , Motoyama, M. , Tagawa, Y. , Akama, K. , Hayashi, T. , Narita, T. , & Chikuni, K. (2017). Qualitative and quantitative comparisons of texture characteristics between broilers and Jidori‐niku, Japanese indigenous chicken meat, assessed by a trained panel. Journal of Poultry Science, 54, 87–96. 10.2141/jpsa.0160066 PMC7477187 32908413

[brb31394-bib-0039] Tomonaga, S. , Noda, K. , Suenaga, R. , Asechi, M. , Adachi, N. , Kino, K. , … Furuse, M. (2007). Stress responses in neonatal meat and layer Nagoya chicks. Animal Science Journal, 78, 541–545. 10.1111/j.1740-0929.2007.00474.x

[brb31394-bib-0040] Trut, L. N. (1999). Early canid domestication: The farm‐fox experiment. American Scientist, 87, 160–169. 10.1511/1999.2.160

[brb31394-bib-0041] Tsudzuki, M. (2003). Japanese native chickens In Chang H. L. & Huang Y. C. (Eds.), The relationship between indigenous animals and humans in APEC region (pp. 91–116). Tainan, Taiwan: CSAS.

[brb31394-bib-0042] Wang, X. , Pipes, L. , Trut, L. N. , Herbeck, Y. , Vladimirova, A. V. , Gulevich, R. G. , … Clark, A. G. (2018). Genomic responses to selection for tame/aggressive behaviors in the silver fox (*Vulpes vulpes*). Proceedings of the National Academy of Science of the United States of America, 115, 10398–10403. 10.1073/pnas.1800889115 PMC6187196 30228118

